# New measure of defensive and constructive optimism towards COVID-19 pandemic

**DOI:** 10.1192/j.eurpsy.2021.759

**Published:** 2021-08-13

**Authors:** T. Gordeeva, O. Sychev

**Affiliations:** 1 Psychology, Lomonosov Moscow State University, Moscow, Russian Federation; 2 Research Department, Shukshin Altai State University for Humanities and Pedagogy, Moscow, Russian Federation; 3 Psychology, National Research University Higher School of Economics and Department of Psychology Moscow State University, Moscow, Russian Federation

**Keywords:** constructive optimism, questionnaire, COVID-19 pandemic, Defensive optimism

## Abstract

**Introduction:**

Individuals’ beliefs about COVID-19 pandemic may affect their health-related behavior including self-isolation. “Positive” beliefs may be realistic (constructive belief that efforts help to prevent infection and spread of the virus) or rose-colored glasses (defensive belief that coronavirus problem is exaggerated aimed to cope with anxiety) with different consequences for behavior and mental health.

**Objectives:**

Objectives: The aim was to develop the scales of defensive and constructive optimism towards COVID-19 pandemic (DCO-Covid) and analyze their psychometric properties, factor structure using CFA, internal consistency and validity.

**Methods:**

The sample comprised 1403 university students (68% women, M=20.59, SD=3.66) from large cities of Russia. Online survey conducted from 10/4/2020 till 25/4/2020. Test battery included the scales of constructive and defensive optimism (each of 3 items) and measure of dispositional optimism (LOT-R, Scheier et al., 1994). Part of the sample (N=306) completed anxiety in a pandemic questionnaire (Tkhostov, Rasskazova, 2020).

**Results:**

CFA indicated a good fit for the two-factor model (χ^2^=27.11, df=8, p<.001, CFI =.985, TLI=.971, RMSEA = .041, p[RMSEA≤.05] = .78) with negative correlations between factors (–.28). Cronbach’s alpha for defensive optimism and constructive optimism were α=.75 and α=.70 respectively. As expected constructive and defensive optimism correlated with dispositional optimism (r=.24; p < .001 and r=–.06; p<.05 respectively) and anxiety (fear of infection, r=.08; n.s and r= –.23; p < .001).

**Conclusions:**

The results show that DCO-C is a reliable measure of defensive and constructive optimism towards COVID-19 pandemic. The construct validity of these scales is confirmed by CFA and obtained correlations.

